# Adenine overload induces ferroptosis in human primary proximal tubular epithelial cells

**DOI:** 10.1038/s41419-022-04527-z

**Published:** 2022-02-02

**Authors:** Muhammad Ali Khan, Purba Nag, Anca Grivei, Kurt T. K. Giuliani, Xiangju Wang, Vishal Diwan, Wendy Hoy, Helen Healy, Glenda Gobe, Andrew J. Kassianos

**Affiliations:** 1 NHMRC CKD CRE (CKD.QLD), University of Queensland, Brisbane, Australia; 2 School of Biomedical Sciences, Faculty of Medicine, University of Queensland, Brisbane, Australia; 3 Conjoint Internal Medicine Laboratory, Chemical Pathology, Pathology Queensland, Brisbane, Australia; 4 Kidney Health Service, Royal Brisbane and Women’s Hospital, Brisbane, Australia; 5 Kidney Disease Research Collaborative, Princess Alexandra Hospital and University of Queensland, Translational Research Institute, Brisbane, Australia; 6 Department of Pharmacy, Bangabandhu Sheikh Mujibur Rahman Science and Technology University, Gopalganj-8100, Dhaka, Bangladesh; 7 Centre for Chronic Disease, Faculty of Medicine, University of Queensland, Brisbane, Australia

**Keywords:** Mechanisms of disease, Interstitial nephritis, Experimental models of disease

## Abstract

The pathogenesis of crystal nephropathy involves deposition of intratubular crystals, tubular obstruction and cell death. The deposition of 8-dihydroxyadenine (DHA) crystals within kidney tubules, for instance, is caused by a hereditary deficiency of adenine phosphoribosyl transferase in humans or adenine overload in preclinical models. However, the downstream pathobiological patterns of tubular cell attrition in adenine/DHA-induced nephropathy remain poorly understood. In this study, we investigated: (i) the modes of adenine-induced tubular cell death in an experimental rat model and in human primary proximal tubular epithelial cells (PTEC); and (ii) the therapeutic effect of the flavonoid baicalein as a novel cell death inhibitor. In a rat model of adenine diet-induced crystal nephropathy, significantly elevated levels of tubular iron deposition and lipid peroxidation (4-hydroxynonenal; 4-HNE) were detected. This phenotype is indicative of ferroptosis, a novel form of regulated necrosis. In cultures of human primary PTEC, adenine overload-induced significantly increased mitochondrial superoxide levels, mitochondrial depolarisation, DNA damage and necrotic cell death compared with untreated PTEC. Molecular interrogation of adenine-stimulated PTEC revealed a significant reduction in the lipid repair enzyme glutathione peroxidase 4 (GPX4) and the significant increase in 4-HNE compared with untreated PTEC, supporting the concept of ferroptotic cell death. Moreover, baicalein treatment inhibited ferroptosis in adenine-stimulated PTEC by selectively modulating the mitochondrial antioxidant enzyme superoxide dismutase 2 (SOD2) and thus, suppressing mitochondrial superoxide production and DNA damage. These data identify ferroptosis as the primary pattern of PTEC necrosis in adenine-induced nephropathy and establish baicalein as a potential therapeutic tool for the clinical management of ferroptosis-associated crystal nephropathies (e.g., DHA nephropathy, oxalate nephropathy).

## Graphical abstract

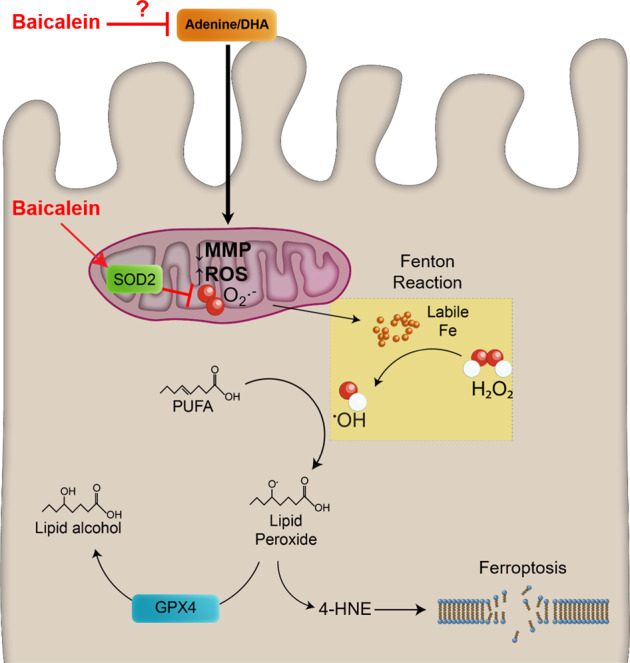

## Introduction

The deposition of crystal microparticles within the kidney promotes tissue damage that can trigger a wide range of acute and/or chronic disorders, collectively termed crystal nephropathies [1]. These crystals are derived from an array of endogenous factors (e.g. minerals, metabolites and proteins) or exogenous substances (e.g. dietary components and drug metabolites) [2]. Emerging evidence indicates that different crystals may, in fact, trigger common pathobiological mechanisms within the kidney [1, 3, 4], thus, paving the way for single targeted therapeutics that treat a broad range of crystal nephropathies.

Of specific interest are the shared cellular and molecular pathways induced in tubular crystallopathies. Tubular cells are highly susceptible to nephrotoxic injuries, in particular, the metabolically active and energy-intensive proximal tubular epithelial cells (PTEC) [5]. The accumulation of crystal deposits within the tubular epithelium leads to mechanical obstruction and regulated cell death (necrosis) [2]. Three pathways of crystal-induced tubular necrosis have been reported: (i) necroptosis—a programmed cell death following receptor-interacting protein kinase 3 (RIPK3)-mediated phosphorylation of the mixed lineage kinase domain-like protein (MLKL) [6]; (ii) mitochondrial permeability transition (MPT)-regulated necrosis—with peptidyl-prolyl isomerase F (PPIF)-dependent formation and permanent opening of the MPT pore [7]; and (iii) ferroptosis—an iron- and reactive oxygen species (ROS)-dependent necrosis mediated by reduced glutathione peroxidase 4 (GPX4) activity and characterised by increased lipid peroxidation (4-hydroxynonenal; 4-HNE) [8]. Necroptosis has been identified as a pattern of in vitro tubular cell death in response to calcium oxalate (CaOx), monosodium urate, calcium phosphate, calcium pyrophosphate dihydrate, cystine and cholesterol crystals [3, 4], whilst both MPT-regulated necrosis and necroptosis contribute to the tubular injury in mice with CaOx-induced acute kidney injury (AKI) and human acute oxalate nephropathy [9]. Moreover, CaOx and folic acid crystals trigger tubular cell ferroptosis in animal models of crystal-induced AKI [10, 11]. However, these emerging mechanistic concepts have yet to be translated to other less common yet clinically challenging crystal nephropathies (e.g. 2,8-dihydroxyadenine/DHA nephropathy).

DHA crystal nephropathy is caused by a deficiency in adenine phosphoribosyl transferase (APRT), an autosomal recessive disorder of purine metabolism [12]. The APRT enzyme catalyses the conversion of adenine derived from polyamine biosynthesis and dietary sources to adenosine monophosphate [4]. However, individuals lacking functional APRT accumulate excess adenine, which is rapidly oxidised to DHA by xanthine dehydrogenase (XDH). The low solubility of DHA results in crystal precipitation within the tubular compartment and recurrent episodes of AKI with the transition to chronic kidney disease (CKD) [13]. The prognosis for patients with APRT deficiency is highly variable, with reports of progression to kidney failure and requirement for renal replacement therapy in more than 20% of cases [14]. Standard therapy includes XDH inhibition with allopurinol or febuxostat, high fluid intake and low purine diet [14, 15]. However, under-dosing of XDH inhibitors and patient nonadherence to long-term treatment contribute to recurrent AKI episodes and CKD progression [4, 16], highlighting a current clinical challenge. Therapeutic targeting of the downstream molecular pathways triggered by DHA crystals (e.g. tubular necrosis) may provide novel treatment options for patients with APRT deficiency.

DHA crystal nephropathy can also be driven directly by adenine overload, as demonstrated in preclinical models of adenine-induced nephropathy. The adenine-enriched diet model in mice and rats displays identical histopathological features (e.g. crystal deposition, tubular injury) to those observed in APRT-deficient patients [17], confirming the clinical relevance of the experimental system for investigating mechanisms of human DHA crystal nephropathy. Of note, crystal deposition and tubular injury in this rodent model are mostly localised to the proximal tubular compartment [17]. However, no studies have explored the patterns of PTEC cell death induced by adenine overload in murine or human DHA crystal nephropathy.

Here, we examine the molecular pathways of adenine-induced tubular necrosis both in an experimental rat model and in human primary PTEC. We establish ferroptosis as the predominant form of regulated cell death in adenine/DHA-induced nephropathy and identify the flavonoid baicalein as a novel therapeutic agent for ferroptosis-associated crystal nephropathies (e.g. DHA-, CaOx-, folic acid-induced nephropathies).

## Results

### Adenine overload induces tubular crystal deposition and ferroptotic cell death in vivo

**Fig. 1:**
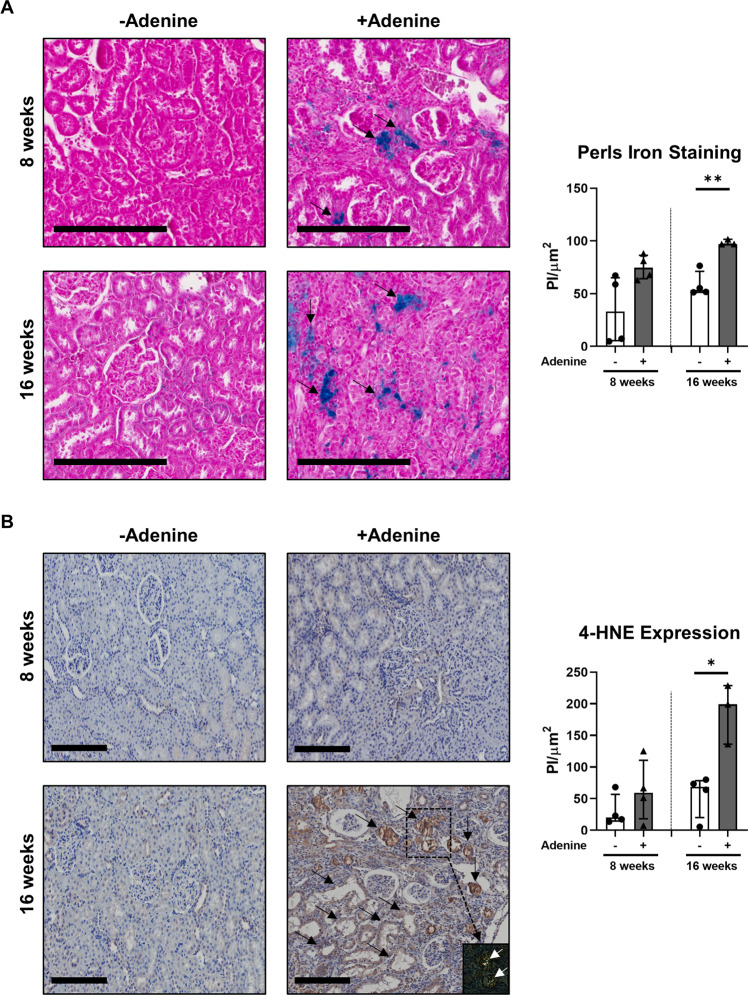
Adenine overload leads to tubular crystal deposition and ferroptotic cell death in rats. Wistar rats (*n* = 3–4/group) were fed powdered rat food alone (-adenine) or powdered food with 0.25% adenine (+adenine) for 8 and 16 weeks. **A** Left panel: Rat kidney sections were assessed for intracellular iron (Perls iron stain - blue) with neutral fast red counterstain (nuclei – red/cytoplasm – pink). Scale bars represent 200 μm. Tubular/peri-tubular positivity is highlighted with black arrows. Right panel: Quantitative analysis (pixel intensity (PI)/µm^2^ area) of Perls staining from three randomly selected areas for each tissue sample is presented. Bar graphs represent median values with interquartile range. *p < 0.05, Welch’s *t*-test. **B** Left panel: IHC labelling of rat kidney sections for lipid peroxidation end-product 4-HNE under light microscopy and with polarisation (inset for adenine-fed rat at 16 weeks) to visualise birefringent crystals (highlighted with white arrows). Scale bars represent 200 μm. Tubular 4-HNE positivity is highlighted with black arrows. Right panel: Quantitative analysis (PI/µm^2^ area) of IHC staining from eight randomly selected areas for each tissue sample is presented. Bar graphs represent median values with interquartile range. *p < 0.05, Welch’s t-test.

We have previously established a rat model of adenine-induced nephropathy [18, 19], characterised by loss of kidney function (proteinuria and increased blood urea nitrogen, plasma uric acid and plasma creatinine) and tubular injury (increased number of dilated tubules, increased cellular debris in the tubular lumen and increased fibrosis) (Supplementary Table 1). Formalin-fixed, paraffin-embedded kidney tissue sections from these previous studies were repurposed in this present investigation to examine the pathobiological pathways of tubular cell death induced by the adenine-enriched diet (0.25%). Adenine overload in rats triggered progressive tubular damage over 16 weeks (Fig. 1A). These structural changes were accompanied by: (i) tubular iron accumulation, with significantly elevated intracellular iron in adenine-fed rats at 16 weeks (Fig. 1A); (ii) tubular lipid peroxidation, with significantly increased 4-HNE immunohistochemical (IHC) staining in the adenine-enriched diet cohort at 16 weeks (Fig. 1B); and (iii) marked deposition of birefringent crystals localised to sites of tubular injury (Fig. 1B). Collectively, these data indicate that adenine-induced crystal cytotoxicity is mediated in vivo via a pathway of tubular ferroptosis, a regulated form of necrosis characterised by the accumulation of iron-dependent lipid peroxides [8].

### Adenine overload selectively induces ferroptotic cell death in human primary PTEC

**Fig. 2:**
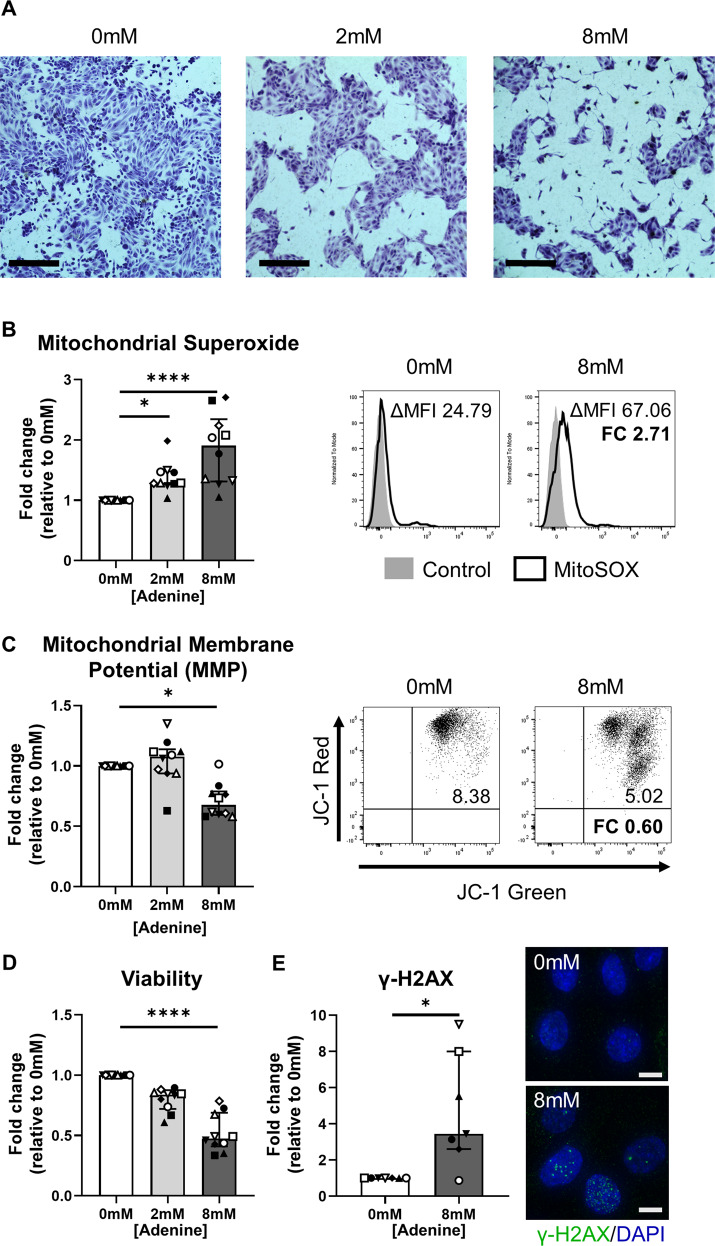
Adenine overload induces pathways of mitochondrial oxidative damage/dysfunction and DNA damage in human primary PTEC. **A** Light microscopy images of PTEC cultured for 48 h in the absence (0 mM) or presence of low-dose (2 mM) and high-dose (8 mM) adenine. Morphology was assessed by toluidine blue staining. PTEC were fixed in 10% buffered formalin solution for 5 min, then stained with 1% toluidine blue solution for 20 min. Scale bars represent 200 μm. One representative of two PTEC donor experiments. **B** Left panel: Fold changes (relative to 0 mM adenine) in mitochondrial superoxide levels [measured as delta median fluorescence intensity (ΔMFI; MFI test – MFI unstained control)] for PTEC cultured in the absence (0 mM) or presence of low-dose (2 mM) and high-dose (8 mM) adenine. Bar graphs represent median values with interquartile range. Symbols represent individual donor PTEC; *n* = 10. *p < 0.05, ****p < 0.0001, Friedman test with Dunn’s post-test. Right panel: Representative MitoSOX staining (black unfilled) compared with unstained control (grey filled) for PTEC cultured in the absence (0 mM) and presence (8 mM) of adenine. Mitochondrial superoxide levels (ΔMFI) are presented for each histogram, with fold change (FC) value relative to 0 mM adenine also shown. **C** Left panel: Fold changes (relative to 0 mM adenine) in mitochondrial membrane potential (MMP) [measured as the ratio of ΔMFI JC-1 red/ΔMFI JC-1 green] for PTEC cultured in the absence (0 mM) or presence of low-dose (2 mM) and high-dose (8 mM) adenine. Bar graphs represent median values with interquartile range. Symbols represent individual donor PTEC; *n* = 10. *p < 0.05, Friedman test with Dunn’s post-test. Right panel: Representative JC-1 dot plots of PTEC cultured in the absence (0 mM) and presence (8 mM) of adenine. MMP values are presented for each histogram, with fold change (FC) value relative to 0 mM adenine also shown. **D** Fold changes (relative to 0 mM adenine) in cell viability (MTT assay) for PTEC cultured in the absence (0 mM) or presence of low-dose (2 mM) and high-dose (8 mM) adenine. Bar graphs represent median values with interquartile range. Symbols represent individual donor PTEC; *n* = 10. ****p < 0.0001, Friedman test with Dunn’s post-test. **E** Left panel: Fold changes (relative to 0 mM adenine) in γ-H2AX protein expression (measured as % cells with >5 γ-H2AX foci) for PTEC cultured in the absence (0 mM) and presence (8 mM) of adenine. Bar graphs represent median values with interquartile range. Symbols represent individual donor PTEC; *n* = 7. *p < 0.05, Wilcoxon matched-pairs signed-rank test. Right panel: Immunofluorescent labelling of representative PTEC cultured in the absence (0 mM) and presence (8 mM) of adenine and stained for γ-H2AX (green) and DAPI (blue). Scale bars represent 20 μm.

To translate our in vivo data to a human system, we performed equivalent in vitro experiments with human primary PTEC, the tubular cell population previously implicated in rodent DHA crystal nephropathy [17]. Human primary PTEC were cultured in the absence (0 mM adenine) or presence of low-dose (2 mM) and high-dose (8 mM) adenine. Examination of PTEC morphology using toluidine blue staining revealed a dose-dependent cytotoxic effect, with 8 mM adenine inducing prominent cell damage/loss (Fig. 2A). Pathways of mitochondrial oxidative stress/dysfunction leading to DNA damage have been previously identified in rat models of adenine-induced nephropathy [20, 21]. In our human system, PTEC treatment with both 2 mM and 8 mM adenine induced significantly elevated mitochondrial superoxide (O_2_ ∙ ^-^) levels compared with control conditions (0 mM adenine) (Fig. 2B). However, only 8 mM adenine significantly reduced PTEC mitochondrial function (↓ mitochondrial membrane potential; MMP) (Fig. 2C) and cell viability (Fig. 2D) compared with control conditions. Moreover, PTEC expression of the DNA damage marker γ-H2AX was significantly increased in response to 8 mM adenine compared with control conditions (Fig. 2E). Thus, subsequent investigations of adenine-induced PTEC injury/death were performed in these high-dose (8 mM) conditions. These collective data identify common pathobiological pathways in adenine-induced human primary PTEC to those identified in rodent models, validating the in vitro system for downstream molecular interrogation of tubular cell death.

**Fig. 3:**
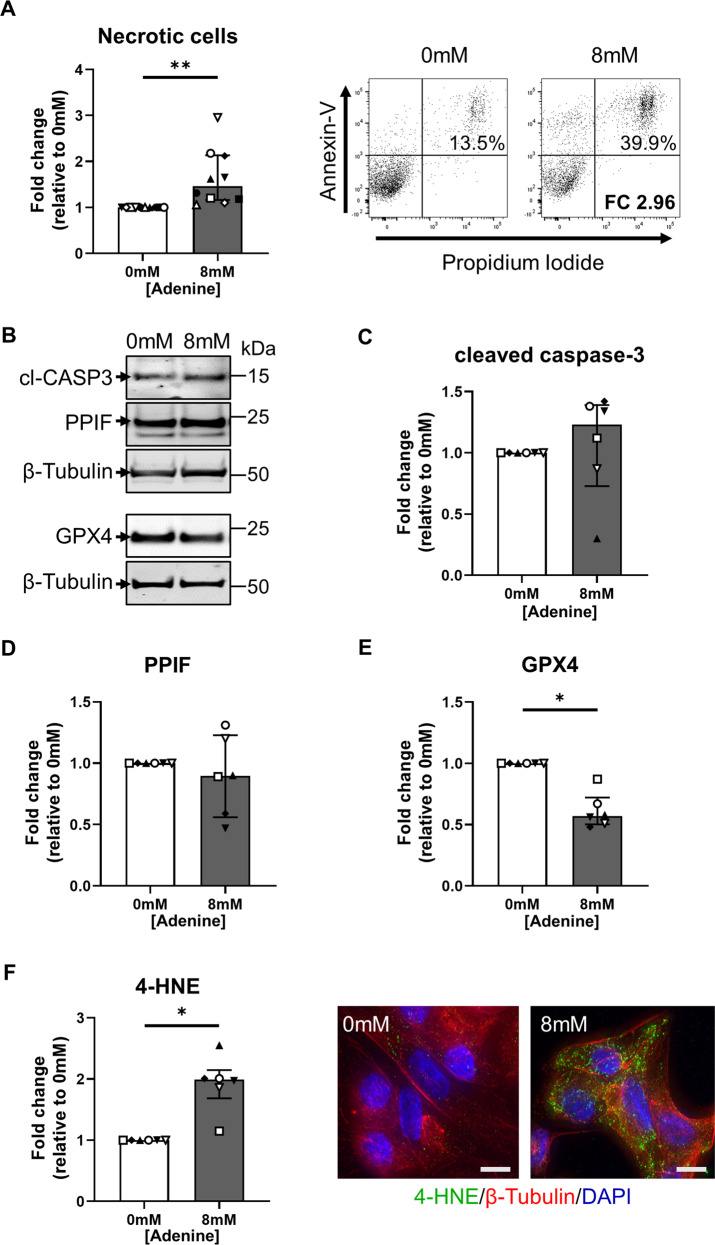
Adenine overload selectively induces ferroptosis in human primary PTEC. **A** Left panel: Fold changes (relative to 0 mM adenine) in cellular necrosis (% Annexin-V^+^ PI^+^ cells) for PTEC cultured in the absence (0 mM) and presence (8 mM) of adenine. Bar graphs represent median values with interquartile range. Symbols represent individual donor PTEC; *n* = 10. **p < 0.01, Wilcoxon matched-pairs signed-rank test. Right panel: Representative donor Annexin-V/PI dot plots of PTEC cultured in the absence (0 mM) and presence (8 mM) of adenine. The percentage of Annexin-V^+^ PI^+^ necrotic cells for each dot plot are presented, with fold change (FC) value relative to 0 mM adenine also shown. **B** Western blot for cleaved caspase-3 (cl-CASP3), PPIF and GPX4 for PTEC cultured in the absence (0 mM) and presence (8 mM) of adenine (15 µg total protein per lane). Representative images from one of six donor PTEC are presented. **C–E** Fold changes (relative to 0 mM adenine) in cleaved caspase 3 **(C)**, PPIF **(D)** and GPX4 **(E)** protein levels (as a ratio of loading control β-tubulin) for PTEC cultured in the absence (0 mM) and presence (8 mM) of adenine. Bar graphs represent median values with interquartile range. Symbols represent individual donor PTEC; n = 6. *p < 0.05, Wilcoxon matched-pairs signed-rank test. **F** Left panel: Fold changes (relative to 0 mM adenine) in 4-HNE expression (measured as mean CTCF of >50 cells per condition) for PTEC cultured in the absence (0 mM) and presence (8 mM) of adenine. Bar graphs represent median values with interquartile range. Symbols represent individual donor PTEC; *n* = 6. *p < 0.05, Wilcoxon matched-pairs signed-rank test. Right panel: Immunofluorescent labelling of representative PTEC cultured in the absence (0 mM) and presence (8 mM) of adenine and stained for 4-HNE (green), β-tubulin (red) and DAPI (blue). Scale bars represent 20 μm.

PTEC death was examined by Annexin-V/propidium iodide (PI) staining. We detected significantly increased cellular necrosis (% Annexin-V^+^ PI^+^ cells) in adenine-stimulated PTEC compared with control PTEC (Fig. 3A). The distinct molecular pathways of cell death were subsequently assessed. Expression levels of apoptosis marker cleaved caspase-3 (Fig. 3B, C) and MPT-regulated necrosis marker PPIF (Fig. 3B, D) were comparable between adenine-stimulated and control PTEC, whilst the necroptosis marker phosphorylated MLKL was not detectable in either PTEC population (data not shown). In contrast, we identified significantly reduced levels of the lipid repair enzyme GPX4 (Fig. 3B, E) and significantly increased 4-HNE expression (Fig. 3F) in adenine-stimulated PTEC compared with control PTEC. Decreased GPX4 and increased 4-HNE are both hallmarks of ferroptotic cell death. Of note, similar levels of PTEC death were observed following adenine overload compared with stimulation using ferroptosis inducer erastin (Supplementary Fig. 1). These data corroborate our in vivo data in establishing ferroptosis as the primary mode of tubular cell death induced by adenine overload.

### Baicalein attenuates adenine-induced ferroptotic death in human primary PTEC via the induction of mitochondrial superoxide dismutase 2 (SOD2)

**Fig. 4:**
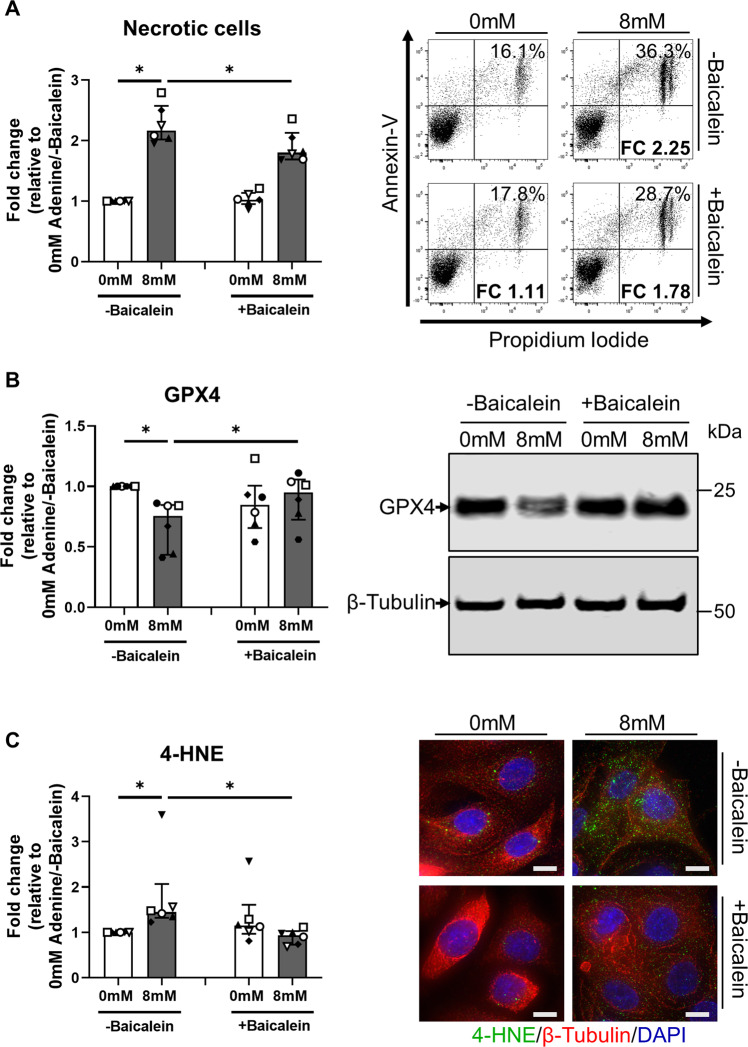
Baicalein attenuates adenine-induced ferroptotic cell death in human primary PTEC. **A** Left panel: Fold changes (relative to 0 mM adenine without baicalein treatment) in cellular necrosis (% Annexin-V^+^ PI^+^ cells) for PTEC cultured in the absence (0 mM) and presence (8 mM) of adenine, both without (-baicalein) and with (+baicalein) treatment. Bar graphs represent median values with interquartile range. Symbols represent individual donor PTEC; *n* = 6. *p < 0.05, Wilcoxon matched-pairs signed-rank test. Right panel: Representative donor Annexin-V/PI dot plots of PTEC cultured in the absence (0 mM) and presence (8 mM) of adenine, both without (-baicalein) and with (+baicalein) treatment. The percentage of Annexin-V^+^ PI^+^ necrotic cells for each dot plot are presented, with fold change (FC) values relative to 0 mM adenine without baicalein treatment also shown. **B** Left panel**:** Fold changes (relative to 0 mM adenine without baicalein treatment) in GPX4 expression for PTEC cultured in the absence (0 mM) and presence (8 mM) of adenine, both without (-baicalein) and with (+baicalein) treatment. Bar graphs represent median values with interquartile range. Symbols represent individual donor PTEC; *n* = 6. *p < 0.05, Wilcoxon matched-pairs signed-rank test. Right panel: Representative GPX4 Western blot for PTEC cultured in the absence (0 mM) and presence (8 mM) of adenine, both without (-baicalein) and with (+baicalein) treatment (15 µg total protein per lane). **C** Left panel: Fold changes (relative to 0 mM adenine without baicalein treatment) in 4-HNE expression (measured as mean CTCF of >50 cells per condition) for PTEC cultured in the absence (0 mM) and presence (8 mM) of adenine, both without (-baicalein) and with (+baicalein) treatment. Bar graphs represent median values with interquartile range. Symbols represent individual donor PTEC; *n* = 6. *p < 0.05, Wilcoxon matched-pairs signed-rank test. Right panel: Immunofluorescent microscopy of representative PTEC cultured in the absence (0 mM) and presence (8 mM) of adenine, both without (-baicalein) and with (+baicalein) treatment, and stained for 4-HNE (green), β-tubulin (red) and DAPI (blue). Scale bars represent 20 μm.

Baicalein [5,6,7-trihydroxyflavone], a flavonoid from the roots of *Scutellaria baicalensis* and *Scutellaria lateriflora*, is a novel pharmacological inhibitor of ferroptosis [22]. In line with this antiferroptotic function, baicalein treatment significantly attenuated cellular necrosis (Fig. 4A), restored GPX4 levels (Fig. 4B) and reduced 4-HNE expression (Fig. 4C) in adenine-stimulated PTEC compared with PTEC having only adenine stimulation. Of note, the protective activity of baicalein in adenine-stimulated PTEC was comparable to that of established ferroptosis inhibitor ferrostatin-1, whilst also demonstrating the capacity to inhibit cell death in erastin-stimulated PTEC (Supplementary Fig. 2).

**Fig. 5:**
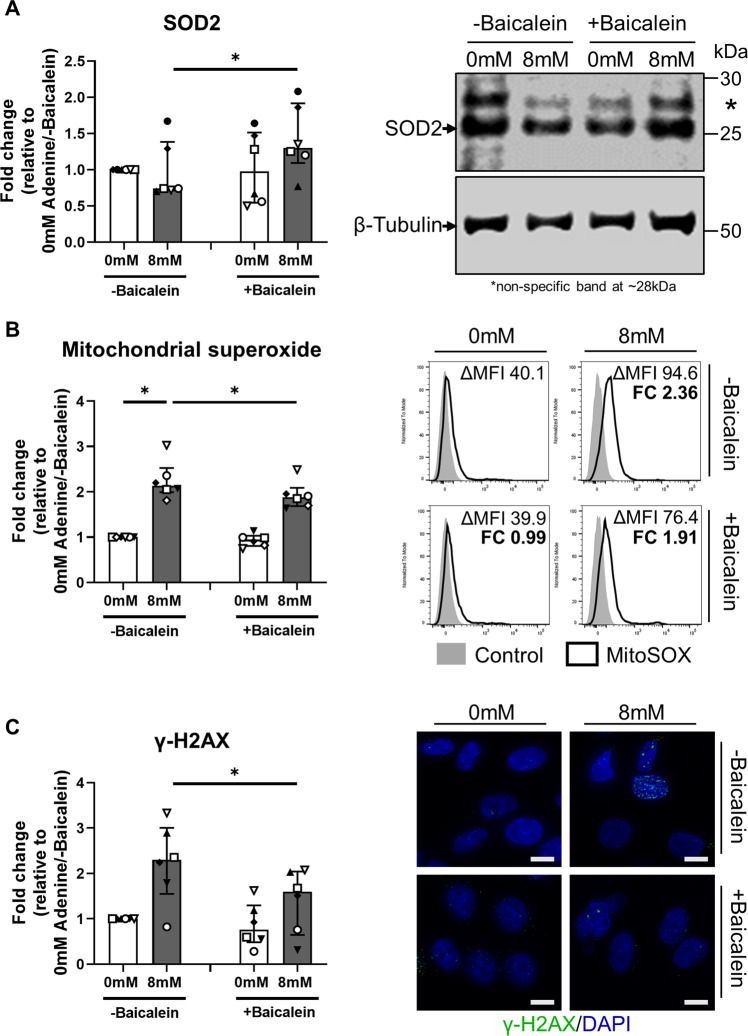
Baicalein attenuates adenine-induced ferroptotic cell death in human primary PTEC via induction of SOD2. **A** Left panel: Fold changes (relative to 0 mM adenine without baicalein treatment) in SOD2 expression for PTEC cultured in the absence (0 mM) and presence (8 mM) of adenine, both without (-baicalein) and with (+baicalein) treatment. Bar graphs represent median values with interquartile range. Symbols represent individual donor PTEC; *n* = 6. *p < 0.05, Wilcoxon matched-pairs signed-rank test. Right panel: Representative SOD2 Western blot for PTEC cultured in the absence (0 mM) and presence (8 mM) of adenine, both without (-baicalein) and with (+baicalein) treatment (15 µg total protein per lane). **B** Left panel: Fold changes (relative to 0 mM adenine without baicalein treatment) in mitochondrial superoxide levels (measured as ΔMFI) for PTEC cultured in the absence (0 mM) and presence (8 mM) of adenine, both without (-baicalein) and with (+baicalein) treatment. Bar graphs represent median values with interquartile range. Symbols represent individual donor PTEC; *n* = 6. *p < 0.05, Wilcoxon matched-pairs signed-rank test. Right panel: Representative MitoSOX staining (black unfilled) compared with unstained control (grey filled) for PTEC cultured in the absence (0 mM) and presence (8 mM) of adenine, both without (-baicalein) and with (+baicalein) treatment. Mitochondrial superoxide levels (ΔMFI) are presented for each histogram, with fold change (FC) value relative to 0 mM adenine without baicalein treatment also shown. **C** Left panel: Fold changes (relative to 0 mM adenine without baicalein treatment) in γ-H2AX expression (measured as % cells with >5 γ-H2AX foci) for PTEC cultured in the absence (0 mM) and presence (8 mM) of adenine, both without (-baicalein) and with (+baicalein) treatment. Bar graphs represent median values with interquartile range. Symbols represent individual donor PTEC; *n* = 6. *p < 0.05, Wilcoxon matched-pairs signed-rank test. Right panel: Immunofluorescent microscopy of representative PTEC cultured in the absence (0 mM) and presence (8 mM) of adenine, both without (-baicalein) and with (+baicalein) treatment, and stained for γ-H2AX (green) and DAPI (blue). Scale bars represent 20μm.

The accumulation of ROS molecules, in particular superoxide generation, is central in ferroptotic cell death [23]. SOD2 is the key antioxidant enzyme responsible for the removal of mitochondrial superoxide radicals [24]. Mechanistic investigations in our human model showed that baicalein treatment significantly increased SOD2 levels in adenine-stimulated PTEC compared with PTEC stimulated only with adenine (Fig. 5A). Demonstrating a functional role for SOD2, baicalein treatment also significantly attenuated levels of mitochondrial superoxide (Fig. 5B) and DNA damage (↓ γ-H2AX) (Fig. 5C) in adenine-stimulated PTEC compared with PTEC stimulated only with adenine. These data highlight a pathobiological function for mitochondrial superoxide in adenine-induced PTEC death. Treatment with baicalein abrogates this pathway of tubular ferroptosis, with potential therapeutic applications for patients with DHA nephropathy.

## Discussion

Crystal deposits of diverse origin can trigger common molecular mechanisms of tubular cytotoxicity [1]. These pathways of tubular injury include ferroptosis, a novel pattern of regulated cell death previously reported in experimental models of CaOx nephropathy and folic acid-induced AKI [10, 11]. In this study, we establish for the first time that ferroptosis also plays a key role in adenine/DHA-induced nephropathy. Moreover, in contrast to other crystal nephropathies where multiple cell death pathways can co-exist [9], our human PTEC data demonstrate the selective induction of ferroptotic cell death in response to adenine overload. Furthermore, we confirm the involvement of this pattern of regulated necrosis via the addition of ferroptosis inhibitor baicalein, thus identifying this plant-based compound as a novel clinical candidate for the treatment of ferroptosis-associated crystal nephropathies.

The adenine-enriched diet model in rodents is a translationally relevant experimental system for examining the pathobiological processes observed clinically in APRT-deficient patients with DHA nephropathy [17]. Indeed, we previously reported that treatment of adenine-fed rats with allopurinol, a XDH inhibitor used as a first-line clinical therapy for human DHA nephropathy, decreased tubular injury, reduced oxidative stress and improved kidney function in rodents [25]. Here, we examine the molecular pathways of tubular injury/oxidative stress in this rat model of adenine-induced nephropathy, demonstrating crystal deposition adjacent to pathological features of tubular iron accumulation and lipid peroxidation (↑ 4-HNE), consistent with a ferroptotic pattern of cell death.

To dissect the mechanisms driving this tubular ferroptosis, we established an in vitro model of adenine overload in human primary PTEC. The epithelial cells of the proximal tubule are of particular interest in crystal nephropathies, including DHA nephropathy, as the primary site of reabsorption from the glomerular filtrate and thus, internalisation and accumulation of intratubular crystals [1, 17]. To enable this energy-intensive reabsorptive function, PTEC have the highest mitochondrial density of any cell within the kidney [26]. Notably, up to 20–50% of total intracellular iron can be found in mitochondrial proteins and is essential to support energy production via oxidative phosphorylation [27]. However, mitochondrial damage can lead to increased free/labile iron that is extremely redox active, catalysing the formation of highly toxic free radicals—hydroxyl radicals (OH∙)—via the Fenton reaction [28]. In turn, free radicals react with polyunsaturated fatty acids (PUFA) to generate lipid peroxides [29]. Lipid repair enzyme GPX4 can neutralise these lipid peroxides by reducing them to their alcohol form [30]. However, if GPX4 function is attenuated, lipid peroxides and their degradation products (e.g. 4-HNE) accumulate to initiate ferroptotic cell death [31]. Although unable to observe detectable crystal deposition in our cultured cells lacking in vivo tubular structure or detect robust intracellular iron in adenine-induced human PTEC by Perls staining (data not shown), we provide first human evidence associating adenine overload in PTEC with these collective pathways of mitochondrial dysfunction (↑ mitochondrial superoxide, ↓ MMP) and ferroptotic cell death (↑ Annexin-V^+^ PI^+^ cells, ↓ GPX4, ↑ 4-HNE).

The flavonoid baicalein is a potent inhibitor of ferroptosis [22]. In our present study, baicalein treatment of adenine-stimulated PTEC restored GPX4 expression, in turn attenuating lipid peroxidation and cellular necrosis. Supporting this concept, previous studies have demonstrated that baicalein blocks ferroptosis by suppressing GPX4 degradation [22, 32]. Alternative antioxidative mechanisms of baicalein-mediated protection have also been identified, including SOD2 activation [32]. SOD2 is the central antioxidant enzyme responsible for reducing superoxide radicals (O_2_ ∙ ^-^) in mitochondria [24]. In our human PTEC model of adenine overload, baicalein-mediated SOD2 induction inhibited ferroptotic cell death by attenuating mitochondrial superoxide levels. This is in line with previous reports that ROS, in particular superoxide radicals, are critical inducers of ferroptotic cell death [8, 33]. Indeed, recent findings in human cancer cells indicate a positive feedback loop between mitochondrial superoxide generation and iron accumulation as the trigger for ferroptosis signalling [33]. In addition to the antioxidative properties of baicalein, a direct inhibitory effect of the flavonoid on xanthine oxidase/XDH has also been reported [34]. We propose that the protective function of baicalein may also be mediated via direct XDH inhibition, thus preventing the oxidation of excess adenine to DHA.

In addition to crystal nephropathies (e.g. calcium oxalate nephropathy and folic acid-induced AKI [10, 11]), ferroptotic tubular cell death has also been reported in: (i) experimental AKI models of ischaemia-reperfusion injury [10, 35], cisplatin-induced nephropathy [36] and rhabdomyolysis [37]; (ii) mouse models of diabetic nephropathy [38]; and (iii) human kidney biopsy specimens of diabetic nephropathy, IgA nephropathy, membranous glomerulonephritis and acute tubular injury patients [38, 39]. Ferrostatin-1, a potent small-molecule compound that inhibits lipid peroxidation [40], has traditionally been used in these experimental models to target ferroptotic cell death [41]. However, the clinical translation of ferrostatin-1 has been questioned due to its poor metabolic stability in vivo [42]. Of note, our data support the previous findings of Xie et al. showing that baicalein exhibits equivalent antiferroptotic activity compared with ferrostatin-1 [22]. Thus, we propose baicalein as a superior drug candidate for the clinical treatment of ferroptosis-associated crystal nephropathies, including DHA nephropathy, but also for the array of AKI and CKD nephropathies associated with ferroptotic tubular cell death. Further validation of baicalein activity in the rat model of adenine-induced nephropathy, including in vivo comparisons to established ferroptosis inhibitors (i.e. ferrostatin-1 or UAMC-3203), will be the critical next step towards such clinical translation.

Collectively, these results provide the first comprehensive molecular characterisation of tubular cell death in adenine/DHA-induced nephropathy. We identify the pathobiological mechanism of tubular ferroptosis in an experimental rat model of adenine-induced nephropathy and in adenine-stimulated human primary PTEC. Moreover, we demonstrate that baicalein attenuates PTEC necrosis in this human preclinical model by targeting upstream regulators of the ferroptotic cascade, including pharmacological modulation of the SOD2 signalling pathway. Our study provides the proof of concept for repurposing of baicalein, currently under investigation in a Phase II influenza clinical trial (NCT03830684), for the therapeutic treatment of ferroptosis-related crystal nephropathies and other renal pathological conditions.

## Materials and methods

### Rat model of adenine-induced nephropathy

All experimental protocols were approved by the Animal Experimentation Ethics Committee of the University of Southern Queensland (10REA419, 11REA006), under the guidelines of the National Health and Medical Research Council of Australia. Male Wistar rats aged 9–10 weeks were treated for up to 16 weeks with powdered rat food as controls or powdered food with 0.25% adenine (Carbosynth Limited, Compton, Berkshire, UK) to achieve a dose of ~150 mg adenine/kg/day. No method of randomisation was used to allocate animals to experimental groups. The dose and delivery of adenine and sample sizes were selected based on previous studies from our laboratory [18, 19]. Urine, plasma and formalin-fixed, paraffin-embedded kidney tissue sections from these previous studies were repurposed in this present investigation to examine cell death mechanisms.

### Collection of urine and plasma

Following 16 weeks of treatment, rats were kept in metabolic cages for 24 h to collect urine. The urine was used for the estimation of protein by the Bradford method [43]. Before the termination of experiments, rats were anesthetised and blood was collected for plasma. Blood urea nitrogen (BUN) and plasma concentrations of uric acid and creatinine were determined by The University of Queensland Veterinary Pathology Services, using an Olympus AU400 auto-analyser [44].

### Rat histology

Upon euthanasia, kidneys were excised, fixed in 10% buffered formalin solution and embedded in paraffin wax. Thin sections (4μm) were cut and stained with haematoxylin and eosin (H&E) to examine tissue histology or Masson’s trichrome stain for assessment of kidney fibrosis by researchers blinded to the experimental results [44]. An Aperio Scanscope XT histology slide scanner (Leica Biosystems, Mt Waverley, VIC, Australia) was used for imaging, with image morphometry analyzed using ImagePro Plus image analysis software. The mean number of dilated tubules per area and the mean number of tubules with cellular debris per area were calculated from ten randomly selected high-power fields for each H&E-stained section. The mean percentage of fibrosis per area was quantified from ten randomly selected high-power fields for each Masson’s trichrome-stained section.

### Perls iron stain and 4-HNE immunohistochemistry

Formalin-fixed, paraffin-embedded rat kidney sections (6 µm) were stained with Perls solution to detect cellular iron accumulation. Briefly, deparaffinised and rehydrated sections were immersed in Perls solution (10% hydrochloric acid and 5% potassium ferrocyanide) for 20 min, then counterstained with nuclear fast red for 5 min.

For IHC examination, paraffin-embedded 6 µm sections were deparaffinised and rehydrated. Endogenous peroxidase activity was blocked with 1% hydrogen peroxide (H_2_O_2_) for 10 min. Heat-induced antigen retrieval was performed in 0.01 M citrate buffer (pH 6.0) for 5 min at 125 °C. Antigen retrieval was followed by a protein block with 2% bovine serum albumin (BSA) (Sigma-Aldrich, St Louis, MO, USA) for 30 min at room temperature. Sections were probed with anti-4-HNE (1:2000; Goat polyclonal IgG; Cat. No. ab46544; Abcam, Cambridge, MA, USA) at 4 °C overnight. Tissue sections were washed and a goat horseradish peroxidase polymer system (Biocare Medical, Pacheco, CA, USA) was applied according to the manufacturer’s instructions. Peroxidase activity was developed with ImmPACT DAB peroxidase (Vector Laboratories, Burlingame, CA, USA) for 5 min. Sections were lightly counterstained with haematoxylin and mounted using DPX Mounting Media.

Perls iron stain and IHC images were obtained using an Aperio AT Turbo (Leica Biosystems) bright field microscope, with quantitative analysis (pixel intensity/µm^2^ area) undertaken using ImageScope (v12.2.2.5015, Leica Biosystems). Statistical analyses for rat studies were performed using a Welch’s t-test, with *P* values ≤ 0.05 considered statistically significant.

### Isolation and culture of human primary PTEC

Kidney cortical tissue was obtained with informed patient consent from the macroscopically healthy portion of renal cell carcinoma nephrectomies, following approval by the Royal Brisbane and Women’s Hospital Human Research Ethics Committee (2002/011). PTEC were subsequently isolated from these specimens following the method of Glynne and Evans [45] and cultured in Defined Medium (DM) as previously described [46]. All PTEC were used in experiments at passage 4.

### Adenine-induced injury in human primary PTEC

PTEC were seeded (100,000 cells/well in DM) in 24-well flat-bottom plates to allow overnight adherence and then further cultured for 48 h (unless otherwise specified) in fresh DM in the absence (0 mM) of adenine (0.002 M sodium hydroxide (NaOH) vehicle control) or presence of adenine (2 mM or 8 mM in 0.002 M NaOH solution) (Sigma-Aldrich). In selected experiments, PTEC were stimulated with 5 µM erastin (MedChemExpress, Monmouth Junction, NJ, USA) for 48 h as a positive control for ferroptosis induction.

For inhibitor studies, 1 µM baicalein (Sigma-Aldrich), 10 µM ferrostatin-1 (Sigma-Aldrich) or 0.033% dimethyl sulfoxide (DMSO) vehicle control were added for the final 24 h of the treatment period. PTEC were harvested by trypsin treatment and analysed for protein expression by Western blotting. PTEC mitochondrial superoxide levels, mitochondrial function and necrosis were assessed by flow cytometry, with cell acquisition performed on an LSR Fortessa (BD Biosciences, San Jose, CA, USA) and data analyzed with FlowJo software (TreeStar, Ashland, OR, USA).

### Protein expression by Western blotting

PTEC were lysed with 8 M urea buffer (Sigma-Aldrich) and protein content determined using the bicinchoninic acid (BCA) protein assay (Pierce Protein Biology/Thermo Fisher Scientific, Waltham, MA, USA). Polyacrylamide gel electrophoresis (PAGE) was undertaken using standard reagents from Invitrogen/Thermo Fisher Scientific. Samples were denatured for 5 min at 95 °C, loaded onto Bolt™ 4–12% Bis-Tris Plus Gels, run at 200 V for 26 min and transferred to a nitrocellulose membrane at 10 V for 68 min. Membranes were blocked for 1 h at room temperature using Odyssey^®^ blocking buffer (LI-COR, Lincoln, NE, USA) and subsequently probed with primary antibodies (Ab) overnight at 4 °C, including cleaved caspase-3 (Asp175) (1:500; Rabbit polyclonal; Cat. No. 9661; Cell Signalling Technology, Danvers, MA, USA), PPIF (1:500; Rabbit polyclonal IgG; Cat. No. SAB4500035; Sigma-Aldrich), MLKL (phospho S358) (1:500; Rabbit monoclonal IgG; Clone EPR9514; Cat. No. ab187091; Abcam), GPX4 (1:750; Rabbit polyclonal IgG; Cat. No. ab41787; Abcam), SOD2 (1:1000; Mouse monoclonal IgG1; Clone mAbcam 74231; Cat No. ab74231; Abcam) and β-tubulin (1:2000; Rabbit polyclonal IgG; Cat No. ab20775; Abcam). Proteins were visualised with IRDye 800CW goat anti-mouse (1:15,000; Millennium Science, Mulgrave, VIC, Australia) or IRDye 680LT goat anti-rabbit (1:20,000; Millennium Science) using the Odyssey CLX (LI-COR). Quantitative analysis of protein intensities relative to the loading control β-tubulin was performed using Image Studio 5.2 software (LI-COR).

### Mitochondrial superoxide detection

Mitochondrial superoxide levels were evaluated using MitoSOX™ Red (Invitrogen) [47]. Briefly, harvested PTEC were incubated with MitoSOX reagent (1 μM, 37 °C, 30 min), with mitochondrial superoxide levels determined by flow cytometry and expressed as the delta median fluorescence intensity (ΔMFI; MFI test – MFI unstained control) for each sample.

### Assessment of mitochondrial membrane potential (MMP)

MMP assessments were performed using cationic dye JC-1 (Invitrogen) [48, 49]. Briefly, PTEC were treated for 24 h in the absence or presence of adenine. PTEC were harvested and incubated with JC-1 (10 µl of 200 µM stock, 37 °C, 30 min), with JC-1 red fluorescence emission (~590 nm) and green fluorescence emission (~529 nm) detected by flow cytometry. The ΔMFI for JC-1 red and green fluorescence were calculated, with the red/green fluorescence ratio representing the MMP for each sample.

### Annexin-V/Propidium Iodide (PI) necrosis assay

The Annexin-V Detection kit I (BD Biosciences) was used to assess PTEC necrosis. Briefly, harvested PTEC were incubated with Annexin-V FITC and PI in binding buffer for 15 min at room temperature. The percentage of Annexin-V^+^ PI^+^ necrotic cells was determined by flow cytometry.

### Cell viability measurements

Cell viability was investigated using the colorimetric MTT (3-(4,5-dimethylthiazol-2-yl)-2,5-diphenyltetrazolium bromide) Cell Proliferation Assay kit (Molecular Probes, Eugene, OR, USA). PTEC were seeded (20,000 cells/well in DM) in triplicate in 96-well flat-bottom plates to allow overnight adherence and then further cultured for 48 h in fresh DM in the absence (0 mM adenine) or presence of adenine (2 mM or 8 mM). MTT solution (10 µl of 12 mM stock) was added to the wells of PTEC, followed by a 2.5 h incubation at 37 °C. The MTT-containing medium was subsequently removed and DMSO applied to the cells, followed by a 10 min incubation at 37 °C. Absorbance values at 540 nm were determined using a Powerwave X52 microplate reader (BioTek Instruments, Winooski, VT, USA).

### Immunofluorescent studies

PTEC were seeded (100,000 cells/well in DM) on sterile coverslips in 24-well flat-bottom plates to allow overnight adherence and then treated in the absence (0 mM) or presence (8 mM) of adenine for 48 h—with addition of 1 µM baicalein for the final 24 h of the treatment period in defined inhibitor studies. Following the treatment period, the cells were fixed with 4% paraformaldehyde (Sigma-Aldrich) in phosphate-buffered saline (PBS; Life Technologies, Grand Island, NY, USA) at room temperature for 10 min, followed by permeabilisation with 0.5% Triton X-100 (Sigma-Aldrich) at room temperature for 30 min and a protein block with 3% BSA/PBS at room temperature for 60 min. PTEC were probed with primary antibodies against 4-HNE (1:100; Goat polyclonal IgG; Cat. No. ab46544; Abcam), the DNA damage marker γ-H2AX (1:1000; Mouse monoclonal IgG; Clone JBW301; Cat. No. 05–636-I; Merck Millipore, Bayswater, Victoria, Australia) and β-tubulin (1:2000; Rabbit polyclonal IgG; Cat. No. ab20775; Abcam) at 4 °C overnight in a humidified chamber. Coverslips were washed thrice with PBS and secondary antibodies applied. Fluorescent detection was obtained by secondary incubation with Alexa Fluor-488 anti-goat IgG, Alexa Fluor-647 anti-mouse IgG and Alexa Fluor-555 anti-rabbit IgG (1:500; all from Life Technologies) at 37 °C for 30 min under humid conditions. Nuclei were stained with DAPI (1:10,000, Invitrogen). Coverslips were mounted on slides in fluorescence mounting medium (Dako Omnis; Agilent Technologies, Santa Clara, CA, USA). Slides were imaged using a DeltaVision deconvolution microscope (GE Healthcare, Pittsburgh, PA, USA), with image processing and analysis using ImageJ software (NIH, Bethesda, MD, USA).

The percentage of cells containing >5 γ-H2AX foci was manually calculated using ImageJ software, with a minimum of 100 cells per experimental condition used for calculations. To quantify cellular 4-HNE, an outline was drawn around each cell using the β-tubulin channel, with area and the integrated density of 4-HNE fluorescence for each cell measured. In addition, calculations for background mean fluorescence (3–4 per image) were recorded. The corrected total cellular fluorescence (CTCF) for each cell was then calculated using the formula: CTCF = integrated density of selected cell—(area of selected cell x mean fluorescence of background readings) [50]. The mean CTCF of >50 cells per experimental condition was then used for comparisons between samples.

### Statistics

Sample sizes were selected based on previous publications from our laboratory with similar experimental design [51]. Data were normalised as a fold change relative to control conditions (0 mM adenine) for each donor. All statistical tests for human PTEC studies were performed using Prism 7.0 analysis software (GraphPad Software, La Jolla, CA, USA). Comparisons between paired groups were performed using a Wilcoxon matched-pairs signed-rank test and multiple comparisons were performed using a Friedman test with Dunn’s post-test. *P* values ≤ 0.05 were considered statistically significant.

## Supplementary information

Supplementary Figure and Table Legends

Supplementary Table 1

Supplementary Figure 1

Supplementary Figure 2

Reproducibility Checklist

## Data Availability

The datasets generated and/or analysed during this study are available from the corresponding author on reasonable request.
