# Supplementary material for: Adenine overload induces ferroptosis in human primary proximal tubular epithelial cells
Source: Cell Death Dis. 2022 Feb 2;13(2):104. doi: 10.1038/s41419-022-04527-z (PMC8810935; doi:10.1038/s41419-022-04527-z)
Supplement: Supplementary file 1 — Supplementary Figure and Table Legends [file 41419_2022_4527_MOESM1_ESM.docx]

**SUPPLEMENTARY FIGURE AND TABLE LEGENDS**

**Supplementary Table 1.** Kidney functional parameters and histology in 0.25% adenine-fed rats (+adenine) compared with powdered rat food alone control rats (-adenine) at 16 weeks. Data represent mean ± standard error mean (SEM). *p<0.05 vs control, Welch’s t-test.

**Supplementary Figure 1.** **Adenine overload induces similar levels of necrosis in human primary PTEC as erastin.** Left panel: Fold changes (relative to 0mM adenine) in cellular necrosis (% Annexin-V^+^ PI^+^ cells) for PTEC cultured in the absence (0mM) and presence (8mM) of adenine or ferroptosis inducer erastin (5μM). Bar graphs represent median values with interquartile range. Symbols represent individual donor PTEC; n=2. Right panel: Representative donor Annexin-V/PI dot plots. The percentage of Annexin-V^+^ PI^+^ necrotic cells for each dot plot are presented, with fold change (FC) values relative to 0mM adenine also shown.

**Supplementary Figure 2. Baicalein-mediated attenuation of adenine-induced PTEC death is comparable to that of established ferroptosis inhibitor ferrostatin-1.** Left panel: Fold changes (relative to 0mM adenine with DMSO vehicle control treatment) in cellular necrosis (% Annexin-V^+^ PI^+^ cells) for PTEC cultured in the absence (0mM) and presence (8mM) of adenine or ferroptosis inducer erastin (5μM), with baicalein, ferrostatin-1 (Fer-1) or DMSO vehicle control treatment for the final 24 h of the culture period. Bar graphs represent median values with interquartile range. Symbols represent individual donor PTEC; n=2. Right panel: Representative donor Annexin-V/PI dot plots. The percentage of Annexin-V^+^ PI^+^ necrotic cells for each dot plot are presented, with fold change (FC) values relative to 0mM adenine with DMSO vehicle control treatment also shown.
