# Supplementary material for: Adenine overload induces ferroptosis in human primary proximal tubular epithelial cells
Source: Cell Death Dis. 2022 Feb 2;13(2):104. doi: 10.1038/s41419-022-04527-z (PMC8810935; doi:10.1038/s41419-022-04527-z)
Supplement: Supplementary file 2 — Supplementary Table 1 [file 41419_2022_4527_MOESM2_ESM.docx]

**Supplementary Table 1.** Kidney functional parameters and histology in 0.25% adenine-fed rats (+adenine) compared with powdered rat food alone control rats (-adenine) at 16 weeks. Data represent mean ± standard error mean (SEM). *p<0.05 vs control, Welch’s t-test.

| **Parameters** | **Control cohort**  **(-adenine; 16 weeks)** | **Test cohort**  **(+adenine; 16 weeks)** |
| --- | --- | --- |
| Protein in urine (mg/l) | 150±7.4 | 302±19.4^*^ |
| Blood urea nitrogen (mmol/l) | 6±0.6 | 56.6±5.4^*^ |
| Plasma uric acid (μmol/l) | 38±1 | 63±4^*^ |
| Plasma creatinine (μmol/l) | 42±2.8 | 268±23^*^ |
| Dilated tubules/area | 0 | 1.6±0.2^*^ |
| Tubules with cell debris/area | 1±0.1 | 4±0.8^*^ |
| % fibrosis/area | 5±0.5 | 36±2.8^*^ |
|  |  |  |
