# Supplementary figures and images for: Adenine overload induces ferroptosis in human primary proximal tubular epithelial cells
Source: Cell Death Dis. 2022 Feb 2;13(2):104. doi: 10.1038/s41419-022-04527-z (PMC8810935; doi:10.1038/s41419-022-04527-z)

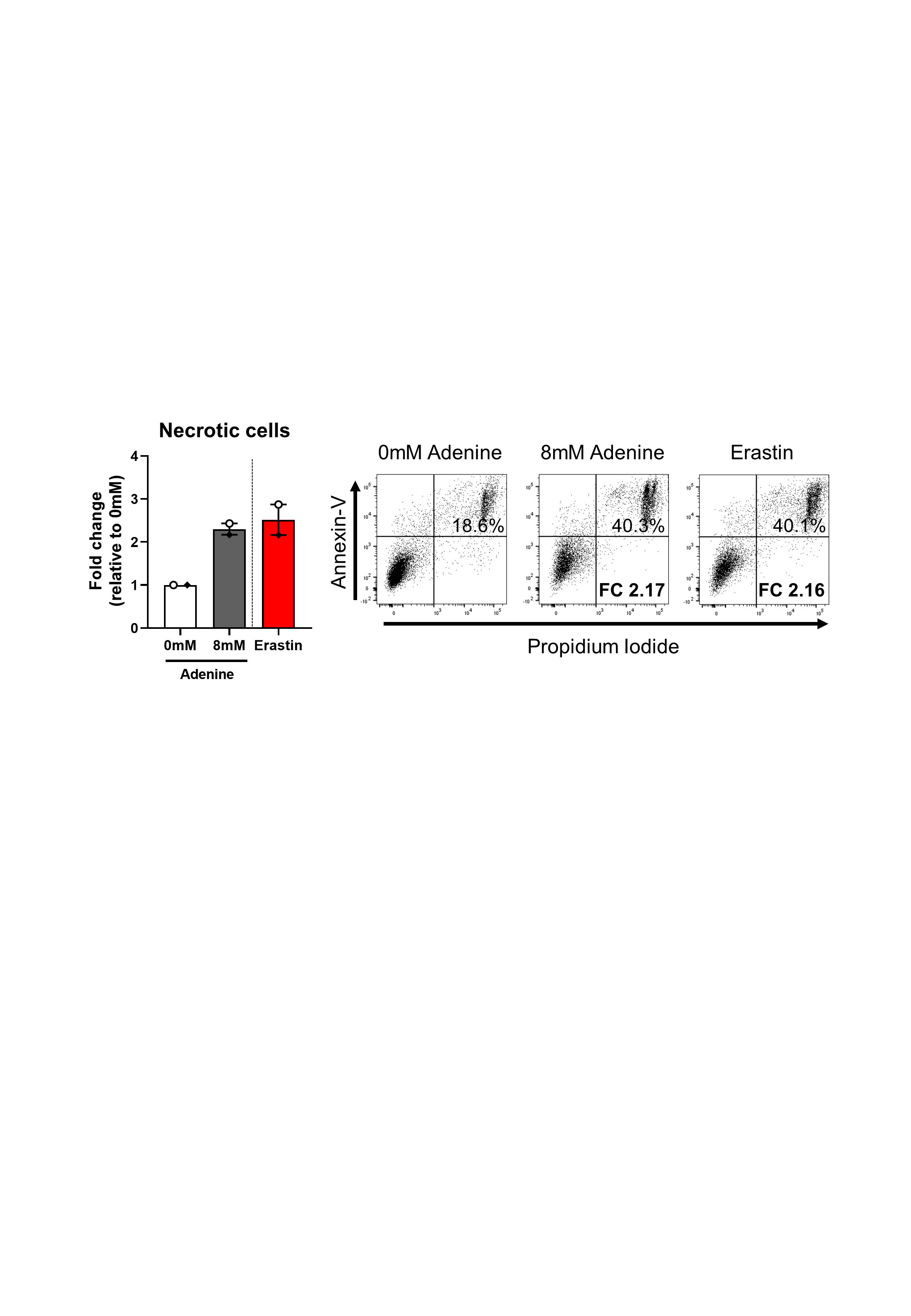

Supplement: Supplementary file 3 — Supplementary Figure 1 [file 41419_2022_4527_MOESM3_ESM.tif]

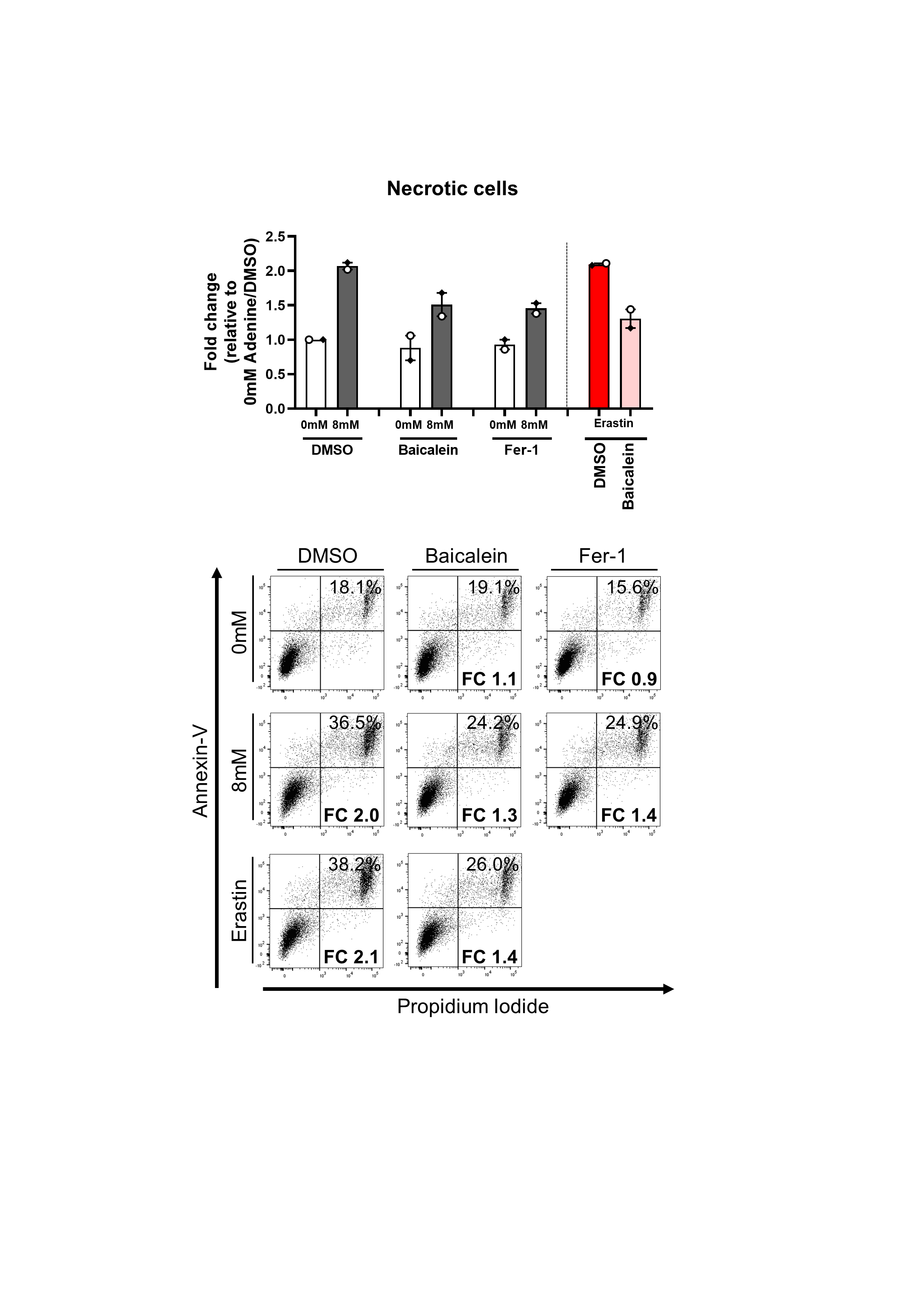

Supplement: Supplementary file 4 — Supplementary Figure 2 [file 41419_2022_4527_MOESM4_ESM.tif]
